# Intelligent Deep-Learning-Enabled Decision-Making Medical System for Pancreatic Tumor Classification on CT Images

**DOI:** 10.3390/healthcare10040677

**Published:** 2022-04-03

**Authors:** Thavavel Vaiyapuri, Ashit Kumar Dutta, I. S. Hephzi Punithavathi, P. Duraipandy, Saud S. Alotaibi, Hadeel Alsolai, Abdullah Mohamed, Hany Mahgoub

**Affiliations:** 1 Department of Computer Sciences, College of Computer Engineering and Sciences, Prince Sattam Bin Abdulaziz University, Al-Kharj 11942, Saudi Arabia; t.thangam@psau.edu.sa; 2 Department of Computer Science and Information Systems, College of Applied Sciences, AlMaarefa University, Ad Diriyah, Riyadh 13713, Saudi Arabia; adotta@mcst.edu.sa; 3 Department of Computer Science and Engineering, Sphoorthy Engineering College, Telangana, Hyderabad 501510, India; hephzi21@gmail.com; 4 Department of Electrical and Electronics Engineering, J. B. Institute of Engineering and Technology, Telangana, Hyderabad 500075, India; vai_2k4@yahoo.co.in; 5 Department of Information Systems, College of Computing and Information System, Umm Al-Qura University, Mecca 21911, Saudi Arabia; sotaibi@uqu.edu.sa; 6 Department of Information Systems, College of Computer and Information Sciences, Princess Nourah Bint Abdulrahman University, P.O. Box 84428, Riyadh 11671, Saudi Arabia; halsolai@pnu.edu.sa; 7 Research Centre, Future University in Egypt, New Cairo, Cairo 11745, Egypt; mohameda@fue.edu.eg; 8 Department of Computer Science, College of Science & Art at Mahayil, King Khalid University, Abha 61421, Saudi Arabia

**Keywords:** decision-making systems, healthcare sector, deep learning, multilevel thresholding, machine learning, artificial intelligence

## Abstract

Decision-making medical systems (DMS) refer to the design of decision techniques in the healthcare sector. They involve a procedure of employing ideas and decisions related to certain processes such as data acquisition, processing, judgment, and conclusion. Pancreatic cancer is a lethal type of cancer, and its prediction is ineffective with current techniques. Automated detection and classification of pancreatic tumors can be provided by the computer-aided diagnosis (CAD) model using radiological images such as computed tomography (CT) and magnetic resonance imaging (MRI). The recently developed machine learning (ML) and deep learning (DL) models can be utilized for the automated and timely detection of pancreatic cancer. In light of this, this article introduces an intelligent deep-learning-enabled decision-making medical system for pancreatic tumor classification (IDLDMS-PTC) using CT images. The major intention of the IDLDMS-PTC technique is to examine the CT images for the existence of pancreatic tumors. The IDLDMS-PTC model derives an emperor penguin optimizer (EPO) with multilevel thresholding (EPO-MLT) technique for pancreatic tumor segmentation. Additionally, the MobileNet model is applied as a feature extractor with optimal auto encoder (AE) for pancreatic tumor classification. In order to optimally adjust the weight and bias values of the AE technique, the multileader optimization (MLO) technique is utilized. The design of the EPO algorithm for optimal threshold selection and the MLO algorithm for parameter tuning shows the novelty. A wide range of simulations was executed on benchmark datasets, and the outcomes reported the promising performance of the IDLDMS-PTC model on the existing methods.

## 1. Introduction

Pancreatic cancer is relatively rare, but it can be a leading cause of death [1,2]. Recently, the survival rate of pancreatic cancer has been low, and its 5-year survival rate gets drastically reduced to 11% [3]. The surgical resection of the primary tumor is possible; in less than 20% of patients, 5-year survival increased to 20–37% [4]. The evidence that impacts survival outcome is sparse and heterogeneous, although delay in diagnoses could also affect the social well-being of patients and their family and quality of life, in part due to the concern that the delay in the number of investigations and consultations would affect treatment prognosis and options [5]. Earlier diagnoses provide better opportunities to reduce the death rate for pancreatic cancer, but a systematic method for earlier diagnoses remains unknown.

Radiomics, as a newly emerging technology, has provided a large amount of data on healthcare images that could expose hidden features of diseases that are observed unclearly with naked eyes. This technique has been investigated to promote and advance cancer management for carcinoma of the lung, colorectal, breast, and bladder to promote and advance cancer management [6]. Furthermore, radiomics extracts a greater number of higher dimension quantitative features from medical images, involving magnetic resonance imaging (MRI), positron emission tomography (PET), and computed tomography (CT). Therefore, CT image is extensively utilized for pancreatic tumor diagnoses. Since roughly 40% of tumors smaller than 2 cm in diameter evade diagnosis by CT, there is a requirement for a new method to increase radiologist analysis in enhancing the sensitivity for the diagnosis of pancreatic cancer [7].

The application of artificial intelligence (AI) to medical diagnostics was initiated in the early 1980s, and computer-assisted diagnosis (CAD) systems with deep learning (DL) have been newly utilized for assisting physicians in enhancing the efficiency of the interpretation of different medical imaging data [8]. The usage of AI in image detection mostly plays two significant roles: computer-assisted diagnosis and computer-assisted detection of lesions for the representation of optical lesions and biopsies. The DL method using convolution neural networks (CNN) has shown considerable potential in analyzing medical images. The neural network construction depends on a stack of neurons comprised of activation functions and parameters to integrate and extract features from the image and establish a model that captures complicated relationships between diagnoses and images [9]. CNN has been reported to accomplish a higher performance in the imaging diagnoses of different conditions involving diabetic retinopathy, liver masses, and skin cancer. However, the potential advantages of CNN for the diagnosis and detection of pancreatic cancer have not been broadly studied [10]. Mostly, pancreatic cancer presents with ill-defined margins and irregular contours on CT and is frequently obscure at an earlier phase, which poses considerable problems even for trained radiotherapists.

This article introduces an intelligent deep-learning-enabled decision-making medical system for pancreatic tumor classification (IDLDMS-PTC) using CT images. The IDLDMS-PTC technique intends to investigate the CT images for the existence of pancreatic tumors. The IDLDMS-PTC model designs an emperor penguin optimizer (EPO) with multilevel thresholding (EPO-MLT) technique for pancreatic tumor segmentation. Additionally, the MobileNet technique was implemented as a feature extractor with optimal auto encoder (AE) for pancreatic tumor classification. In order to optimally adjust the weight and bias values of the AE method, the multileader optimization (MLO) algorithm was utilized. To assess the effectiveness of the IDLDMS-PTC technique, a comprehensive experimental analysis was carried out on a benchmark dataset.

## 2. Literature Review

In Sujatha’s research [11], a different diagnosis technique is presented for the recognition of pancreatic tumors with image texture character that is estimated statistically. Diagnoses were completed by deep wavelet neural networks (DWNN). A DL-based hierarchical CNN (HCNN) is presented for pancreatic tumor diagnoses [12]. The RNN was proposed for meeting the problems of spatial discrepancy segmentation over slices of adjacent images. Liang et al. [13] designed an approach that allows automated segmentation of pancreatic GTV based on multiple parametric MRIs with DNN.

Asadpour et al. [14] presented a cascaded architecture for extracting the tumor in adenocarcinoma patients and the volumetric shape of the pancreas. This method is an integration of an elastic atlas that is able to fit on three-dimensional volumetric shapes extracted from CT slices, a CNN using three forwarded paths to label the patches of the image with coarse to fine resolution with a multi-resolution architecture.

The capacity of DL is estimated in [15] to distinguish pancreatic disease on contrast-enhanced magnetic resonance (MR) images with the help of a generative adversarial network (GAN). Classification accuracy of trained InceptionV4 architecture for each patient and patch were made on the validation set, correspondingly. Iwasa et al. [16] estimated the ability of DL for the automated segmentation of pancreatic tumors on CE-EUS video images and the probable factor that affects the automated segmentation. Automated segmentation was implemented by U-Net with 100 epochs and estimated by using four-fold cross-validation. The tumor boundary (TB) and degree of respiratory movement (RM) were classified as to 3-degree intervals from the patient and estimated as feasible factor that affects the segmentation.

## 3. The Proposed Model

**Figure 1:**
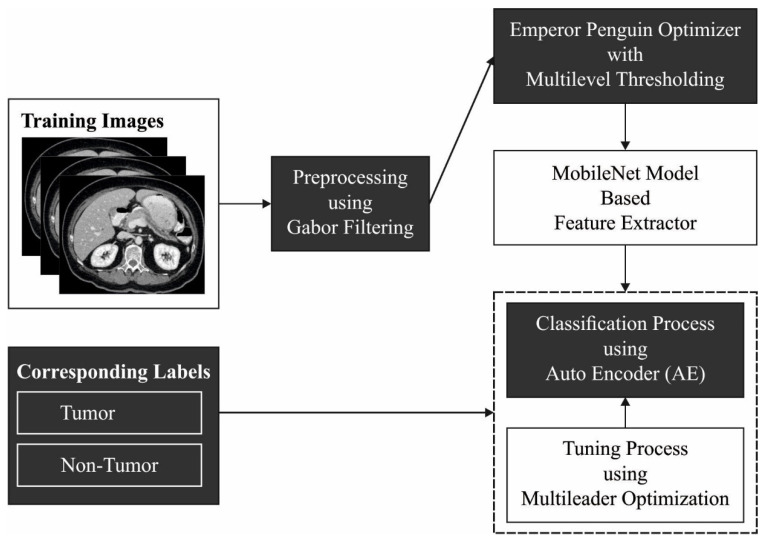
Overall process of IDLDMS-PTC technique.

In this study, a novel IDLDMS-PTC technique was derived from examining the CT images for the existence of pancreatic tumors. The proposed IDLDMS-PTC technique comprises several subprocesses, namely GF-based pre-processing, EPO-MLT-based segmentation, MobileNet-based feature extraction, AE-based classification, and MLO-based parameter optimization. The utilization of the EPO approach to better threshold selection and the MLO algorithm for parameter tuning assists in accomplishing improved classification results. Figure 1 illustrates the overall process of the IDLDMS-PTC technique.

### 3.1. Gabor Filtering Based Pre-Processing

At the initial stage, the pre-processing of the CT images is performed by the use of the GT technique. It is a linear filter whose impulse response is a sinusoidal function multiplied by a Gaussian function. They are nearly passband function. The major benefit of presenting the Gaussian envelope is that the Gabor function is situated in spatial and frequency domains, different from the sinusoidal function, which is entirely delocalized in the spatial (sinusoidal function covers the whole space) and localized accurately in the frequency domain [17]. Thus, this function is highly suited for representing a signal in these domains. Gabor is a 2D bandpass filter; when allocated a frequency and direction, the address of the original image is conserved, and noise is reduced.

### 3.2. EPO-MLT-Based Segmentation

In the image segmentation process, the EPO-MLT approach was developed for determining the tumor regions in the CT images. The MLT issue was demonstrated by assuming a gray level image I that was segmented containing K+1 classes [18]. Therefore, K thresholds $\left\{t_{1},\;t_{2},\;\ldots ,\;t_{K}\right\}$ are needed for dividing the image into sub-regions as in Equation (3):

(1) $$C_{0}=\left\{g\left(u,v\right)\in I|0\leq g\left(u,v\right)\leq t_{1}-1\right\}$$

(2) $$C_{1}=\{g\left(u,v\right)\in I|t_{1}\leq g\left(u,v\right)\leq t_{2}-1\}$$

(3) $$C_{K}=\left\{g\left(u,v\right)\in I|t_{K}\leq g\left(u,v\right)\leq L-1\right\}$$

where $C_{k}$ implies the $k^{th}$ class of images, $t_{k}$ $\left(k=1,\ldots ,K\right)$ refers to the $k^{th}$ threshold values, $g\left(u,v\right)$ stands for the gray level of pixels $\left(u,v\right),$ and L signifies the gray level of I; these levels are from the range $\left(0,1\ldots L-1\right)$. The vital drive of multilevel thresholding is for locating the threshold value that divides pixels into many groups, which are defined as maximized in the subsequent formula:

(4) $$t_{1}^{*},t_{2}^{*},\ldots ,t_{K}^{*}=\underset{t_{1},\ldots ,t_{k}}{\max}\;F\left(t_{1},\ldots ,t_{K}\right)$$

where $F\left(t_{1},\ldots ,t_{K}\right)$ refers to the Otsu’s function, which is determined as:

(5) $$F=\overset{K}{\underset{u=0}{\sum }}A_{u}{\left(\eta_{u}-\eta_{1}\right)}^{2},$$

(6) $$A_{u}=\overset{t_{u+1}-1}{\underset{v=\tau_{u}}{\sum }}P_{v},$$

(7) $$\eta_{u}=\overset{\tau_{u+1}-1}{\underset{v=l_{v}}{\sum }}u\frac{P_{v}}{A_{v}},\;where\;P_{l}=h\left(u\right)/N_{p}$$

where $\eta_{1}$ refers to the mean intensity of I with $t_{0}=0$ and $t_{K+1}=L$. The $h\left(u\right)$ and $P_{u}$ implies the frequency and probability of $u^{th}$ gray level; correspondingly, $N_{p}$ stands for the entire number of pixels from I.

In order to define the optimum threshold values for the MLT approach, the EPO algorithm is applied. The EPO was stimulated by the emperor penguins (EPs) huddling attitudes as initiated in the Antarctic [19]. In order to forage, the EPs usually travel from rafl/colonies. Therefore, an initial function is for defining an effective mover in the swarm from the mathematical progress. The distances amongst Eps $\left(X_{ep}\right)$ were calculated for achieving this, then its temperature profile $\left(\theta^{\prime }\right)$. The temperature profile of EPs is measured as:


(8) $$\theta^{\prime }=\left(\theta -\frac{Iter_{\;\max}}{C-Iter_{\;\max}}\right)$$

(9) $$\theta =\left\{\begin{array}{c} 0\;if\;R>0.5\\ 1\;if\;R<0.5 \end{array}\right.$$

The higher number of iterations, where C implies the existing iteration, was stated as $Iter_{\;\max}$, and R implies the arbitrary number between zero and one.

As EPs usually huddle together to preserve temperature, careful precaution is taken to protect them from neighborhood collisions. Consequently, it presents two vectors $\left(\vec{U}\right)$ and $\left(\vec{V}\right)$ whose values are estimated as:

(10) $$\vec{U}=M\times \left(\theta^{\prime }+X_{grid}\;\left(accuracy\right)\right)\times Rand()-\theta^{\prime }$$

(11) $$\vec{V}=Rand()$$

(12) $$X_{grid}\;\left(accuracy\right)=\left|\vec{X}-\vec{X}\right|$$

where M indicates movement with the fixed value of 2, $\vec{x}$ represents an optimum solution, ${\vec{x}}_{ep}$ signifies places of other EPs, [0, 1], and ‖ defines the absolute value of Rand.


(13) $$\vec{D}=\left|\left\{S(\vec{U})\cdot \vec{X}\left(x\right)-\vec{V}\cdot \vec{X}\left(x\right)\right\}\right|$$

(14) $$S(\vec{U})=\sqrt{{\left(fe^{-C/v}-e^{-C}\right)}^{2}}$$

Equations (13) and (14) are created for calculating approximately the distance among EP and optimally fittest searching agents $\left(\vec{D}\right)$. S() illustrates the social force to that an optimally searching agent was managed by EPs; e represents the exponential functions.

At this point, based on the optimum agents achieved utilizing Equation (15), the places of EPs were upgraded.


(15) $${\vec{X}}_{ep}\left(x+1\right)=\vec{X}\left(x\right)-\vec{U}\cdot {\vec{D}}_{ep}$$

It must be apparent that the parameter range selected is equal to individuals of new works. Therefore, the EPO technique has been employed for obtaining the optimal global value with respect to the operator. In EPO, with arbitrarily created individual EPs, the population of emperor penguins is initialized. 

### 3.3. MobileNet-Based Feature Extraction

At the time of feature extraction, the segmented images are passed into the MobileNet model to generate feature vectors. AI has connected the gap between the abilities of machines and humans. Computer vision is a field of AI that allows machines to observe the world like humans. The advancement in this field has been completed over one certain approach named a CNN. CNN contains input, hidden, and output layers [20]. To design a diagnostic system for pancreatic tumor classification, in this study, the MobileNet model is utilized for feature extraction. MobileNet is faster when compared to convolution networks because of its different filter methods for every response channel. This method is built on depth-wise separable convolution that has two successive functions: one is a depth-wise convolutional at the filter phase, which employs convolutional to a single input channel at a time, and the other is a point-wise convolution at the filtering phase, which implements linear integration of output to the depth-wise convolution. ReLU and Batch normalization (BN) layers come after the convolutional process. Computation cost phenomenally reduces in the depth-wise separable model because of filtration at the integrating phase to minimalize its complexity and size. The version applied here employs MobileNet V2 with 3.47 million parameters.

### 3.4. Optimal AE-Based Classification

Finally, the AE model is used to detect and classify the presence of pancreatic cancer. An AE employs a set of weights recognition for encoding an input vector x into a depiction vector h, represented as latent parameters [21]. Then, it employs a set of weights generative for decoding the depiction vector into an estimated reconstruction of the input vector $x^{\prime }$. The aim of the AE is to recreate the input information in an unsupervised manner, that is, without utilizing any labels when the dimensionality of the input and the output need to be identical.

The encoder phase of AE takes $x\in {\mathbb{R}}^{m}$ as input and maps to latent parameter $h\in {\mathbb{R}}^{n}$:

(16) $$h=f\left(Wx+b\right)$$

where f denotes an activation function, namely sigmoid, $s\left(x\right)=1/1+e^{-x}$ or ReLU, W indicates a weight matrix, and b represents a bias vector. Then, the decoder phase maps h to $x^{\prime }$ that is a reconstruction of x with similar dimensionality.

(17) $$x^{\prime }=f^{\prime }\left(W^{\prime }h+b^{\prime }\right)$$

Here, $f^{\prime },W^{\prime }$, and $b^{\prime }$ indicate the respective parameters for the decoder that may be distinct from the encoder one. AE is trained for minimizing reconstruction errors, including mean squared error (MSE):

(18) $$E\left(x,x^{\prime }\right)={\left\|x-x^{\prime }\right\|}^{2}$$

x is usually averaged through n trained instances. For determining the weight and bias values of the AE technique, the MLO approach can be utilized. It is arithmetically modeled for implementing optimization problems. The major concept of the presented approach is to utilize data from the members of the population. In such cases, member of the population uses the data of different leaders for searching in the problem-solving space as follows:

(19) $$X_{i}=\left[\chi_{i}^{1},\ldots ,\chi_{i}^{d},x_{i}^{m}\right]$$

where $X_{i}$ represent the ith member of the population, and $\chi_{i}^{d}$ indicates the dth parameter of optimization problem. Members of the population are estimated by placing them in the fitness function [22]. Next, the population matrix is arranged according to the minimum values of the fitness function as follows:

(20) $$X^{sort}=\left[\left.\begin{array}{c} X_{1}^{sort}\\ \vdots \\ X_{N}^{sort} \end{array}\right|\left.\begin{array}{c} X_{r_{1}}\\ \vdots \\ X_{r_{N}} \end{array}\right|\begin{array}{c} \min\left(fit\right)\\ \vdots \\ \max\left(fit\right) \end{array}\right]$$

Here, $X^{sort}$ indicates the matrix of population, $X_{1}^{sort}$ represent the member with optimal fitness value, $X_{N}^{sort}$ indicates the member with worst fitness value, $X_{r_{1}}$ shows the member of the population with initial rank-based fitness value, $X_{r_{N}}$ represent the member of the population with worst rank-based fitness value, fir indicates the fitness value, and N signifies the number of members of the population. After arranging the population matrix, some amount of the ranked population is chosen as the leader. The leader is upgraded with all the iterations to guide members of the population towards the optimum solution as follows:

(21) $$L=\left\{X_{l}^{sort},X_{l}^{sort}\in X^{sort},\;l=1:n_{L}\right\}$$

(22) $$x^{sort}={\left[\begin{array}{c} X_{1}^{sort}\\ \vdots \\ X_{n_{L}}^{sort}\\ \vdots \\ X_{N}^{sort} \end{array}\right]}_{N\times m}\to \;L={\left[\begin{array}{c} X_{1}^{sort}\\ \vdots \\ X_{l}^{sort}\\ \vdots \\ X_{n_{L}}^{sort} \end{array}\right]}_{n_{l}\times m}$$

where L indicates the selected leader member matrix and $n_{L}$ shows the leader’s number. The population in MLO is upgraded as follows. Initially, all the members of the population are moved in the searching space according to the leader position. The leader is defined according to the roulette wheel. All the leaders might be chosen for updating different parameters of the presented solution.

(23) $$fit_{i}^{n}=\frac{fit_{i}-max\left(fit\right)}{\Sigma_{j=1}^{N}\left(fit_{j}-max\left(fit\right)\right)}$$

(24) $$P_{l}=\frac{fit_{l}^{n}}{\Sigma_{j=1}^{n_{l}}fit_{j}^{n}}$$

(25) $$C_{l}=P_{l}+C_{l-1},C_{0}=0\;\&\;l=1:n_{l}$$

(26) $$L_{i,c}^{d}=\left\{\begin{array}{cc} L_{1}=X_{1}^{sort}, & 0\leq r\leq C_{1}\\ \vdots & \vdots \\ L_{c}=X_{c}^{sort}, & C_{c-1}\leq r\leq C_{1}\\ \vdots & \vdots \\ L_{n_{l}}=X_{n_{L}}^{sort}, & C_{n_{l}-1}\leq r\leq C_{n_{l}} \end{array}\right.$$

(27) $$\chi_{i,new}^{d}=\chi_{i}^{d}+rand\left(L_{i,c}^{d}-2\times x_{i}^{d}\right)$$

(28) $$X_{i}=\left\{\begin{array}{cc} X_{i,new}, & fit_{i,new}\leq fit_{i}\\ X_{i}, & else \end{array}\right.$$

In the equation, $fit_{i}^{n}$ indicates the normalized fitness function for the ith population member, $P_{l}$ represent the possibility of selection of l’th leader for guiding the parameter, $C_{l}$ indicate the cumulative probability of l’th leader, $x_{i,new}^{d}$ shows the new value for dth dimension of ith parameter, $L_{i,c}^{d}$ denotes the dth dimension of selected cth leader for guiding dth parameter of ith population member, and r shows the arbitrary value within [0, 1]. Next, after upgrading the initial stage, all the members of the population make a slight random move in their own neighborhood. When the new location is highly relevant, the member upgrades its location to new status or else returns to its preceding location.

(29) $$x_{i,new}^{d}=x_{i}^{d}+2\times \left(1-\frac{\tau }{T}\right)\times \left(-0.2+rand\times 0.4\right)\times x_{i}^{d}$$

(30) $$X_{i}=\left\{\begin{array}{c} X_{i,new},\;fit_{i,new}\leq fit_{i}\\ X_{i},\;else \end{array}\right.$$

Here, t denotes the $t^{th}$ iteration, and T represent the maximal number of iterations.

The MLO algorithm has determined a fitness function of tuning the parameter values of the AE model. The fitness function is used to determine a positive integer for the representation of effectual outcomes of the candidate solutions. Here, the objective is to minimize the classification error rate as given below.

(31) $$fitness\left(x_{i}\right)=ClassifierErrorRate\left(x_{i}\right)\;=\frac{number\;of\;misclassified\;images}{Total\;number\;of\;images}*100$$

## 4. Experimental Validation

**Figure 2:**
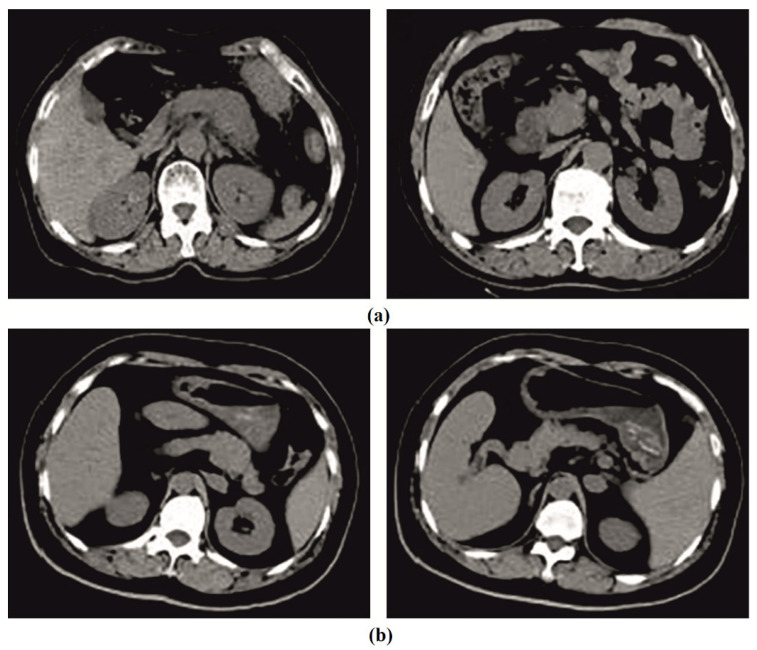
Sample images: (**a**) tumor; (**b**) non-tumor.

This section investigates the pancreatic tumor detection and classification performance of the IDLDMS-PTC model using test CT images collected from various sources. The dataset holds a total of 500 images, with 250 images under pancreatic tumor and 250 images under non-pancreatic tumor. The results are investigated under varying sizes of training/testing datasets. Figure 2 depicts sample CT images.

### 4.1. Results Analysis

**Figure 3:**
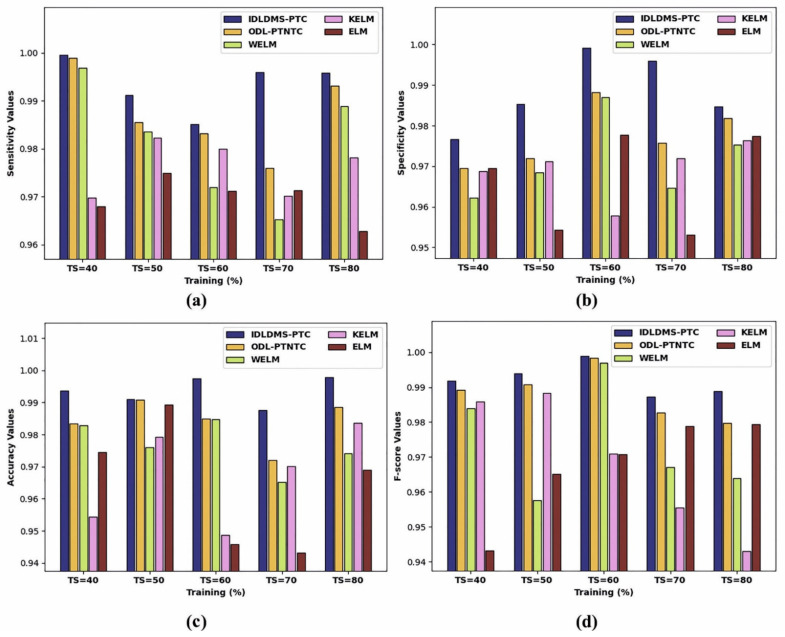
Result analysis of IDLDMS-PTC technique under different sizes of training set: (**a**) sensitivity, (**b**) specificity, (**c**) accuracy, and (**d**) F-score.

Table 1 and Figure 3 report the overall classification outcomes of the IDLDMS-PTC with existing models under varying sizes of training sets (TS). The experimental outcomes indicated that the IDLDMS-PTC model obtained effectual outcomes under distinct TSs. For instance, the IDLDMS-PTC model obtained a higher average $sens_{y}$ of 0.9935 where the ODL-PTNTC, WELM, KELM, and ELM models obtained lower average $sens_{y}$ values of 0.9989, 0.9969, 0.9697, and 0.9679, respectively. Along with that, the IDLDMS-PTC model gained an increased average $spec_{y}$ of 0.9884 where the ODL-PTNTC, WELM, KELM, and ELM models provided reduced average $spec_{y}$ values of 0.9775, 0.9715, 0.9692, and 0.9664, respectively. Furthermore, the IDLDMS-PTC model reached a maximum average $accu_{y}$ of 0.9935 where the ODL-PTNTC, WELM, KELM, and ELM models resulted in minimum average $accu_{y}$ values of 0.9840, 0.9766, 0.9672, and 0.9644, respectively. Moreover, the IDLDMS-PTC model exhibited increased an average $F_{score}$ of 0.9948 where the ODL-PTNTC, WELM, KELM, and ELM models depicted decreased average $F_{score}$ values of 0.9882, 0.9739, 0.9688, and 0.9674, respectively.

**Figure 4:**
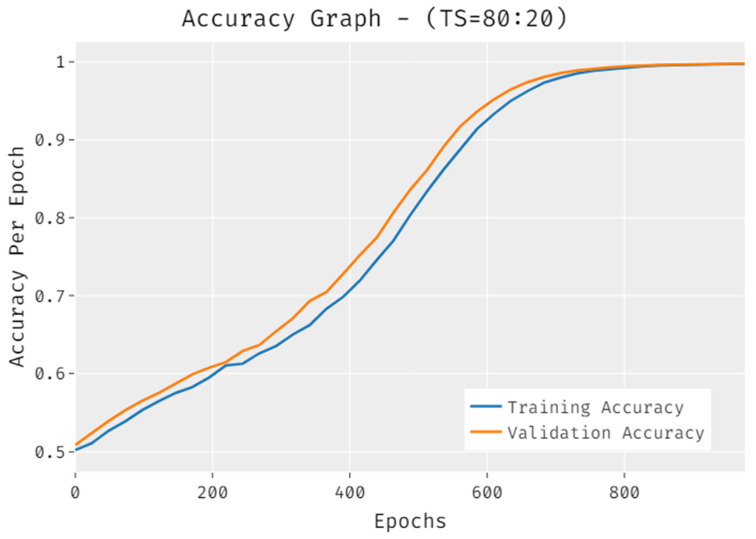
Accuracy of IDLDMS-PTC technique under training set (80:20).

The accuracy of the IDLDMS-PTC approach under training set (80:20) data is portrayed in Figure 4. The results demonstrated that the IDLDMS-PTC technique accomplished improved validation accuracy compared to training accuracy. It can also be observed that the accuracy values get saturated with the count of epochs.

**Figure 5:**
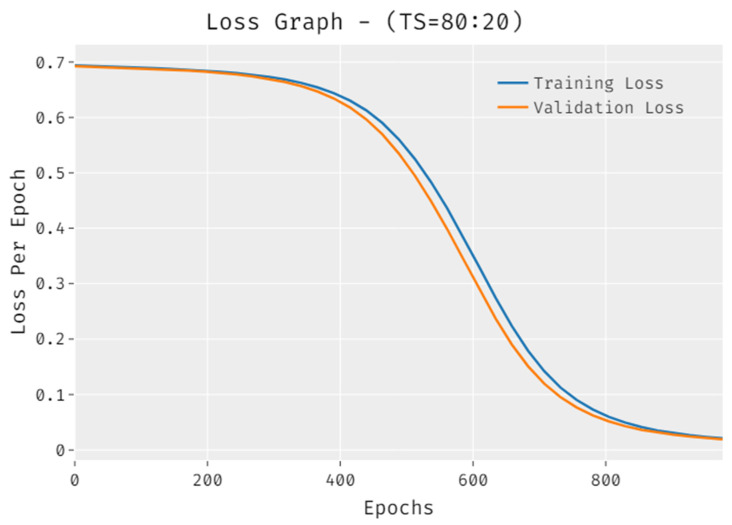
Loss of IDLDMS-PTC technique under training set (80:20).

The loss outcome analysis of the IDLDMS-PTC technique under training set (80:20) data is depicted in Figure 5. The figure exposed that the IDLDMS-PTC technique has denoted the reduced validation loss over the training loss. It can be additionally observed that the loss values get saturated with the count of epochs.

**Figure 6:**
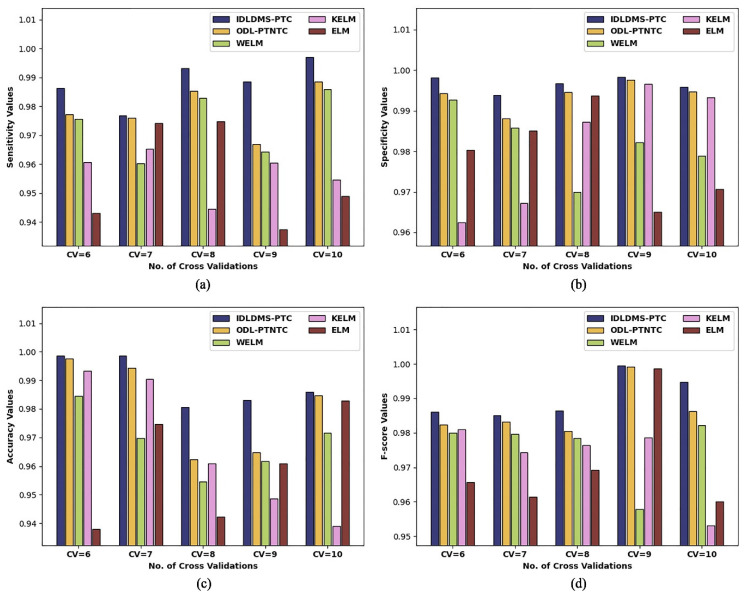
Result analysis of IDLDMS-PTC technique under different sizes of CV: (**a**) sensitivity, (**b**) specificity, (**c**) accuracy, and (**d**) F-score.

Table 2 and Figure 6 report the overall classification outcomes of the IDLDMS-PTC with existing techniques under varying sizes of CV. The experimental outcomes indicate that the IDLDMS-PTC algorithm obtained effectual outcomes under distinct CVs. For instance, the IDLDMS-PTC model obtained a higher average $sens_{y}$ of 0.9884 where the ODL-PTNTC, WELM, KELM, and ELM approaches achieved minimum average $sens_{y}$ values of 0.9788, 0.9738, 0.9571, and 0.9557, respectively. Followed by that, the IDLDMS-PTC model gained an increased average $spec_{y}$ of 0.9965 where the ODL-PTNTC, WELM, KELM, and ELM approaches provided decreased average $spec_{y}$ values of 0.9938, 0.9819, 0.9813, and 0.9789, respectively.

Additionally, the IDLDMS-PTC model reached a maximal average $accu_{y}$ of 0.9894, where the ODL-PTNTC, WELM, KELM, and ELM approaches resulted in minimum average $accu_{y}$ values of 0.9808, 0.9685, 0.9665, and 0.95977, respectively. Moreover, the IDLDMS-PTC model exhibited an increased average $F_{score}$ of 0.9904 where the ODL-PTNTC, WELM, KELM, and ELM models outperformed decreased average $F_{score}$ values of 0.9863, 0.9756, 0.9727, and 0.9710, respectively.

**Figure 7:**
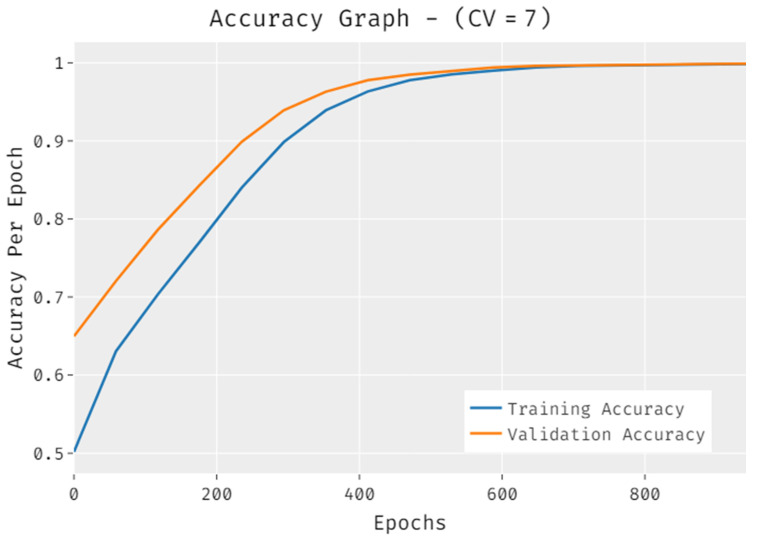
Accuracy analysis of IDLDMS-PTC technique under CV of 7.

The accuracy outcome analysis of the IDLDMS-PTC technique under CV of 7 data is illustrated in Figure 7. The results demonstrate that the IDLDMS-PTC technique accomplished improved validation accuracy compared to training accuracy. It is also observable that the accuracy values get saturated with the epoch count.

**Figure 8:**
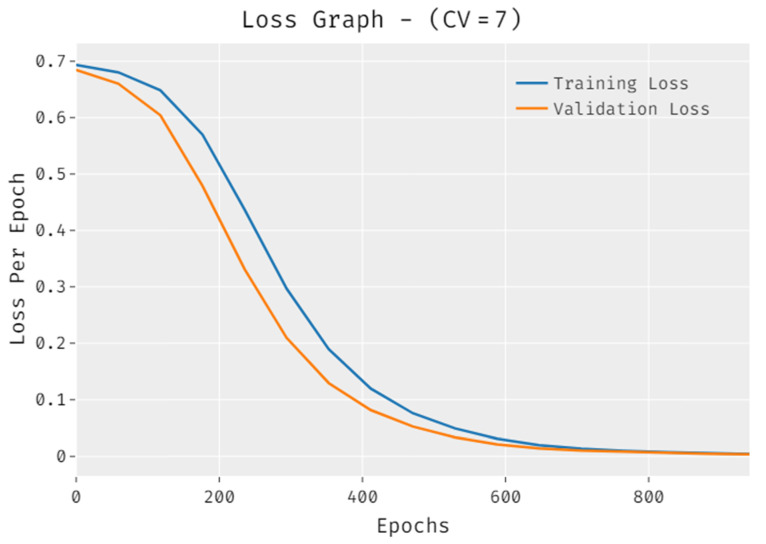
Loss analysis of IDLDMS-PTC technique under CV of 7.

The loss outcome analysis of the IDLDMS-PTC technique under CV of 7 data is displayed in Figure 8. The figure demonstrates that the IDLDMS-PTC methodology signified the reduced validation loss over the training loss. It is additionally noted that the loss values get saturated with the epoch count.

### 4.2. Discussion

**Figure 9:**
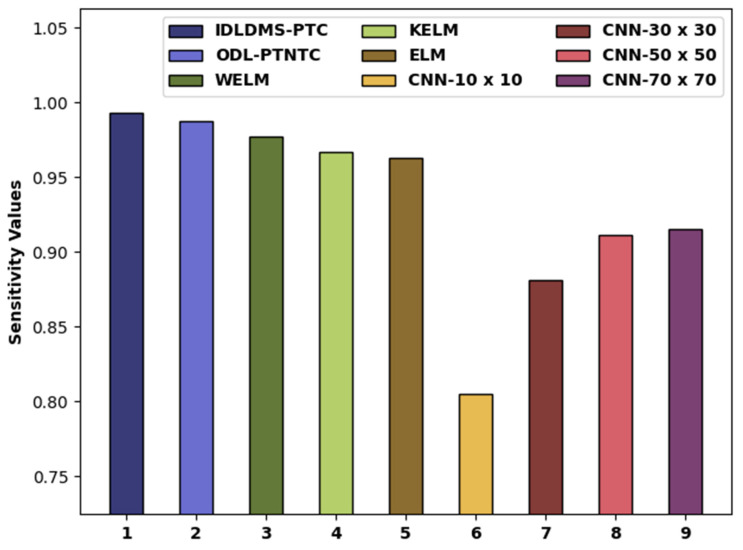
$Sens_{y}$ analysis of IDLDMS-PTC technique compared with recent approaches.

In order to further ensure the betterment of the proposed model, a detailed comparative study of the IDLDMS-PTC approach with recent approaches is offered in Table 3 [23,24,25,26]. Figure 9 portrays the $sens_{y}$ examination of the IDLDMS-PTC technique with existing approaches. The figure defined that the CNN-10x10 and CNN-30x30 models obtained lower $sens_{y}$ values of 0.8050 and 0.8810, respectively. Afterward, the CNN-50x50 and CNN-70x70 techniques attained slightly increased $sens_{y}$ values of 0.9110 and 0.9150, respectively. In line with, the WELM, KELM, and ELM models resulted in moderately closer $sens_{y}$ of 0.9776, 0.9666, and 0.9627, respectively. Though the ODL-PTNTC model accomplished near optimum $sens_{y}$ of 0.9873, the presented IDLDMS-PTC methodology reached a maximum $sens_{y}$ of 0.9935.

**Figure 10:**
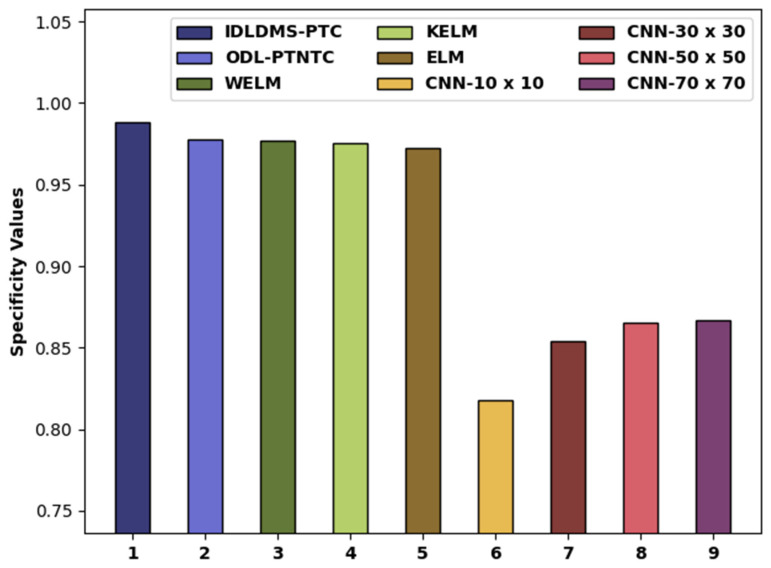
$Spec_{y}$ analysis of IDLDMS-PTC technique compared with recent approaches.

Figure 10 depicts the $spec_{y}$ examination of the IDLDMS-PTC technique with existing approaches. The figure reported that the CNN-10x10 and CNN-30x30 methods obtained lower $spec_{y}$ values of 0.8180 and 0.8540. Likewise, the CNN-50x50 and CNN-70x70 models attained slightly increased $spec_{y}$ values of 0.8650 and 08670, respectively. Moreover, the WELM, KELM, and ELM models resulted in moderately closer $spec_{y}$ of 0.9767, 0.9753, and 0.9727, respectively. Next, the ODL-PTNTC model accomplished near-optimal $spec_{y}$ of 0.9775, and the projected IDLDMS-PTC technique attained increased $spec_{y}$ of 0.9884.

**Figure 11:**
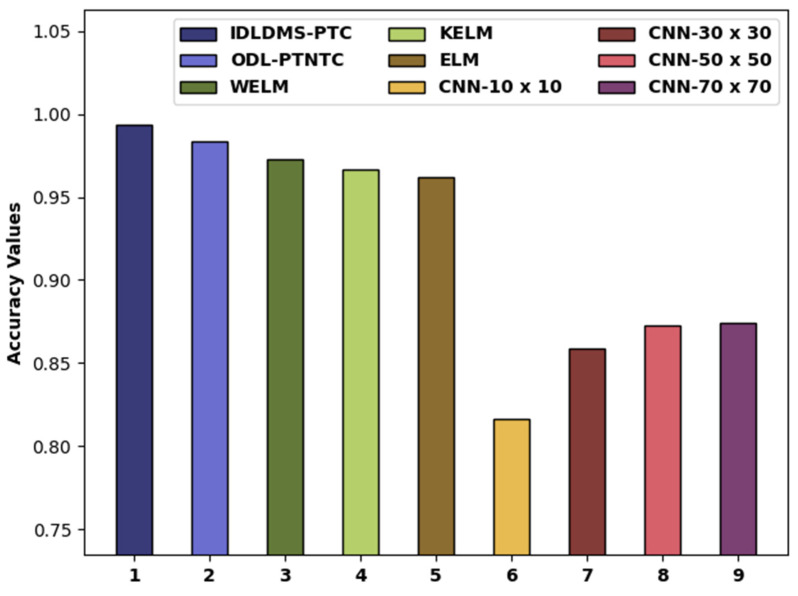
$Acc_{y}$ analysis of IDLDMS-PTC technique compared with recent approaches.

Figure 11 compares the $acc_{y}$ examination of the IDLDMS-PTC system with existing algorithms. The figure revealed that the CNN-10x10 and CNN-30x30 methods obtained lower $acc_{y}$ values of 0.8160 and 0.8590, respectively. Similarly, the CNN-50x50 and CNN-70x70 methods attained slightly increased $acc_{y}$ values of 0.8730 and 0.8740, respectively. Additionally, the WELM, KELM, and ELM algorithms resulted in moderately closer $acc_{y}$ of 0.9726, 0.9669, and 0.9621, respectively. Eventually, the ODL-PTNTC system has accomplished near-optimal $acc_{y}$ of 0.9840, and the presented IDLDMS-PTC technique has reached a maximum $acc_{y}$ of 0.9935.

By looking into the above-mentioned tables and figures, it can be ensured that the IDLDMS-PTC model resulted in superior pancreatic tumor detection and classification performance over the other methods. The reduced network size, minimum parameters, and faster performance of the MobileNet model help in assisting improved performance. Additionally, the utilization of the EPO technique for optimum threshold selection and the MLO algorithm for parameter tuning assists in accomplishing improved classification results.

## 5. Conclusions

In this study, a novel IDLDMS-PTC approach was derived from examining the CT images for the existence of pancreatic tumors. The proposed IDLDMS-PTC technique comprises several subprocesses, namely GF-based pre-processing, EPO-MLT-based segmentation, MobileNet-based feature extraction, AE-based classification, and MLO-based parameter optimization. The utilization of the EPO technique to optimum threshold selection and the MLO algorithm for parameter tuning assists in accomplishing improved classification results. To assess the effectiveness of the IDLDMS-PTC technique, a comprehensive experimental analysis was carried out on a benchmark dataset. Extensive comparative outcomes exposed the promising performance of the IDLDMS-PTC model over the existing methods. Therefore, the IDLDMS-PTC technique can be utilized as an effective tool for the healthcare system. In the future, deep instance segmentation approaches will be applied to improve the classifier results of the IDLDMS-PTC model.

## Figures and Tables

**Table 1:** Results analysis of IDLDMS-PTC technique under different sizes of training set with existing approaches.

Training (%)	IDLDMS-PTC	ODL-PTNTC	WELM	KELM	ELM
Sensitivity					
TS = 40	0.9995	0.9989	0.9969	0.9697	0.9679
TS = 50	0.9912	0.9855	0.9835	0.9823	0.9749
TS = 60	0.9851	0.9832	0.9720	0.9800	0.9712
TS = 70	0.9960	0.9759	0.9653	0.9702	0.9713
TS = 80	0.9958	0.9931	0.9889	0.9782	0.9628
Average	0.9935	0.9873	0.9813	0.9761	0.9696
Specificity					
TS = 40	0.9767	0.9696	0.9622	0.9687	0.9696
TS = 50	0.9853	0.9720	0.9684	0.9712	0.9543
TS = 60	0.9992	0.9882	0.9870	0.9578	0.9778
TS = 70	0.9959	0.9757	0.9646	0.9719	0.9531
TS = 80	0.9847	0.9818	0.9753	0.9764	0.9774
Average	0.9884	0.9775	0.9715	0.9692	0.9664
Accuracy					
TS = 40	0.9937	0.9834	0.9829	0.9544	0.9746
TS = 50	0.9911	0.9908	0.9760	0.9792	0.9893
TS = 60	0.9974	0.9850	0.9847	0.9487	0.9459
TS = 70	0.9876	0.9721	0.9652	0.9702	0.9432
TS = 80	0.9978	0.9886	0.9742	0.9837	0.9690
Average	0.9935	0.9840	0.9766	0.9672	0.9644
F-score					
TS = 40	0.9919	0.9892	0.9839	0.9859	0.9432
TS = 50	0.9940	0.9908	0.9576	0.9884	0.9651
TS = 60	0.9989	0.9984	0.9970	0.9710	0.9708
TS = 70	0.9873	0.9827	0.9671	0.9555	0.9788
TS = 80	0.9889	0.9798	0.9640	0.9431	0.9793
Average	0.9948	0.9882	0.9739	0.9688	0.9674

**Table 2:** Result analysis of IDLDMS-PTC technique under different sizes of CV with existing approaches.

No. of Folds	IDLDMS-PTC	ODL-PTNTC	WELM	KELM	ELM
Sensitivity					
CV=6	0.9864	0.9773	0.9757	0.9607	0.9431
CV=7	0.9768	0.9761	0.9603	0.9654	0.9741
CV=8	0.9931	0.9853	0.9829	0.9445	0.9748
CV=9	0.9885	0.9670	0.9644	0.9605	0.9375
CV=10	0.9970	0.9885	0.9859	0.9546	0.9489
Average	0.9884	0.9788	0.9738	0.9571	0.9557
Specificity					
CV = 6	0.9981	0.9942	0.9927	0.9625	0.9803
CV = 7	0.9938	0.9880	0.9857	0.9672	0.9851
CV = 8	0.9967	0.9945	0.9699	0.9872	0.9937
CV = 9	0.9983	0.9975	0.9822	0.9965	0.9650
CV = 10	0.9958	0.9947	0.9788	0.9932	0.9706
Average	0.9965	0.9938	0.9819	0.9813	0.9789
Accuracy					
CV = 6	0.9986	0.9977	0.9846	0.9934	0.9379
CV = 7	0.9987	0.9944	0.9698	0.9904	0.9746
CV = 8	0.9807	0.9623	0.9546	0.9610	0.9422
CV = 9	0.9831	0.9648	0.9618	0.9486	0.9609
CV = 10	0.9860	0.9847	0.9715	0.9389	0.9829
Average	0.9894	0.9808	0.9685	0.9665	0.9597
F-score					
CV = 6	0.9861	0.9824	0.9799	0.9810	0.9657
CV = 7	0.9850	0.9832	0.9796	0.9744	0.9615
CV = 8	0.9864	0.9805	0.9784	0.9764	0.9692
CV = 9	0.9995	0.9992	0.9578	0.9786	0.9987
CV = 10	0.9948	0.9863	0.9821	0.9531	0.9600
Average	0.9904	0.9863	0.9756	0.9727	0.9710

**Table 3:** Comparative analysis of IDLDMS-PTC technique with recent approaches.

Methods	Sensitivity	Specificity	Accuracy
IDLDMS-PTC	0.9935	0.9884	0.9935
ODL-PTNTC	0.9873	0.9775	0.9840
WELM	0.9776	0.9767	0.9726
KELM	0.9666	0.9753	0.9669
ELM	0.9627	0.9727	0.9621
CNN-10x10	0.8050	0.8180	0.8160
CNN-30x30	0.8810	0.8540	0.8590
CNN-50x50	0.9110	0.8650	0.8730
CNN-70x70	0.9150	0.8670	0.8740

## Data Availability

Data sharing is not applicable to this article, as no datasets were generated during the current study.
